# Different responses of fish/microbial transglutaminase to salt and ultrasound: Implications for myosin cross-linking

**DOI:** 10.1016/j.ultsonch.2025.107723

**Published:** 2025-12-13

**Authors:** Xia Gao, Meng Gui, Gang Yu, Yongqiang Zhao, Liang Gao, Ru Liu

**Affiliations:** a Fisheries Sciences Institute, Beijing Academy of Agriculture and Forestry Sciences, National R&D Branch Center for Conventional Freshwater Fish Processing (Beijing), Beijing 100068, PR China; b Key Laboratory of Efficient Utilization and Processing of Marine Fishery Resources of Hainan Province, Sanya Tropical Fisheries Research Institute, Sanya 572426, PR China; c Key Laboratory of Aquatic Product Processing, Ministry of Agriculture and Rural Affairs of the People's Republic of China, National R&D Center for Aquatic Product Processing, South China Sea Fisheries Research Institute, Chinese Academy of Fisheries Sciences, Guangzhou, Guangdong 510300, PR China; d College of Food Science and Technology, Huazhong Agricultural University, Engineering Research Center of Green Development for Conventional Aquatic Biological Industry in the Yangtze River Economic Belt, Ministry of Education, National R&D Branch Center for Conventional Freshwater Fish Processing (Wuhan), Wuhan, Hubei 430070, PR China

**Keywords:** Fish transglutaminase, Microbial transglutaminase, Salt concentrations, High intensity ultrasound, Myosin cross-linking, Water holding capacity

## Abstract

This study investigated the differential responses of fish transglutaminase (FTGase) and microbial transglutaminase (MTGase) to NaCl concentrations and high intensity ultrasound (HIU) during myosin cross-linking. Increasing NaCl concentrations enhanced MTGase activity by 34.5% at 0.8 mol/L compared to the control without NaCl. This enhancement was accompanied by structural unfolding, as evidenced by increased UV absorption intensity, which indicated greater exposure of aromatic residues. In contrast, FTGase activity progressively declined with increasing NaCl concentrations, with minimal structural changes observed. Given its Ca^2+^-independent property, MTGase was used to explore the synergistic effect of HIU and NaCl. The combination of 400 W HIU and 0.3 mol/L NaCl induced the most pronounced structural changes in MTGase, which exposed some buried reactive sulfhydryl groups and elevated activity by 17.0%. Furthermore, HIU pretreatment of both enzymes enhanced their catalytic efficiency for myosin cross-linking, as evidenced by increased formation of ε-(γ-Glu)-Lys isopeptide bonds. Notably, while HIU-pretreated FTGase improved the water holding capacity (WHC) of myosin samples, HIU-pretreated MTGase likely induced excessive cross-linking, which paradoxically reduced WHC. Conversely, applying HIU directly to preformed enzyme-myosin complexes disrupted established cross-links. These findings provide a basis for optimizing transglutaminase applications in food processing using HIU.

## 1 Introduction

Transglutaminase (TGase) is a critical cross-linking enzyme extensively utilized to enhance the textural properties (such as hardness, gumminess, and chewiness), water holding capacity (WHC), and microstructures of surimi gels by catalyzing the formation of ε-(γ-Glu)-Lys isopeptide bonds between glutamine and lysine residues [1,2]. Microbial TGase (MTGase) has been reported to strengthen the gel properties in diverse surimi systems, including those from flying fish, hairtail, sardine, white croaker, and spotted golden goatfish [[2], [3], [4], [5], [6], [7]]. There is endogenous TGase in fish surimi (FTGase), which is also responsible for setting and gel network formation of surimi gels [8]. Notably, FTGase and MTGase exhibit fundamental structural and functional differences that arise from their distinct biological origins. A typical distinction lies in their Ca^2+^-dependence: FTGase requires Ca^2+^ for activation, whereas MTGase is typically Ca^2+^-independent [9]. These inherent structural divergences suggest differential responses to processing environments, including ionic strength and physical fields.

NaCl plays an indispensable dual role in surimi gelation, as it solubilizes myosin: the primary functional protein responsible for forming the foundational gel network that dictates key textural properties, and it modulates TGase activity [[10], [11], [12], [13]]. However, there is a knowledge gap regarding the comparative responses of FTGase and MTGase to varying NaCl concentrations under identical conditions, especially considering their contrasting Ca^2+^ requirements.

Currently, the growing demand for healthier foods has intensified interest in salt-reduced surimi products [14,15]. While MTGase can partially compensate for impaired gel properties in salt-reduced surimi [16], our prior work demonstrated that high intensity ultrasound (HIU) improved the gel properties of salt-reduced (1.5% NaCl) surimi, and this effect was linked to enhanced FTGase activity [17]. Besides, HIU has been reported to inhibit protein and lipid oxidation, improve whiteness, and facilitate the formation of denser microstructures of surimi gels [[18], [19], [20]]. Subsequent investigations confirmed that HIU activated both FTGase and MTGase, with a significantly stronger activation observed for MTGase [21]. These findings suggested a potential synergistic interaction between HIU and NaCl in modulating TGase activity; however, the underlying mechanism remains to be elucidated.

This study systematically compared the enzymatic activity response of FTGase and MTGase to NaCl concentrations and elucidated associated structural changes using UV spectroscopy. Meanwhile, molecular dynamics simulations were used to probe the structural basis of Ca^2+^-dependence in FTGase. Furthermore, the synergistic effect of HIU and NaCl on MTGase activity and structure was investigated. Subsequently, the catalytic efficiency of FTGase and MTGase in cross-linking myosin under HIU was analyzed in terms of aggregate behavior, chemical bonds, and WHC. This study will provide insights that are essential for optimizing TGase utilization, particularly in developing high-quality salt-reduced surimi products.

## 2 Materials and methods

### 2.1 Materials

Silver carp (*Hypophthalmichthys molitrix*, 1.5–2.0 kg body weight) was purchased from a local market in Wuhan, China, and transported live to the laboratory under chilled conditions within 30 min. *N, N*-Dimethyl casein (DMC), monodansylcadaverine (MDC), and adenosine 5′-triphosphate disodium salt hydrate (ATP-Na_2_) were obtained from Sigma-Aldrich Chemical Co. (St. Louis, MO, USA). MTGase (3000 U/g) was supplied by Kinetika Biotechnical Co. (Lugano, Switzerland). All other chemicals were of analytical grade and acquired from Sinopharm Chemical Reagent Co., Ltd. (Shanghai, China).

### 2.2 Preparation of samples

#### 2.2.1 Extraction of FTGase, myosin, and preparation of MTGase solution

FTGase was isolated using the method of Worratao & Yongsawatdigul [22]. Briefly, minced meat was mixed with extraction buffer (60 mmol/L Tris, 40 mmol/L NaCl, 5 mmol/L EDTA, and 2 mmol/L DTT, pH 7.5) at a 1:3 (w/v) ratio. Following homogenization, the mixture was held at 4 °C for 30 min and centrifuged at 12,000 rpm for 15 min. The supernatant obtained after centrifugation constituted the crude enzyme extract.

To prepare the MTGase solution, 0.500 g of MTGase was accurately weighed out and solubilized in 100 mL of Tris-HCl buffer (pH 7.5). The solution was thoroughly mixed and stored at 4 °C for further analysis. FTGase and MTGase samples were adjusted with NaCl to a series of salt concentrations.

The myosin extraction procedure followed the method described by Liu et al. [23] with slight modifications. Briefly, minced fresh fish meat was homogenized in buffer (0.1 mol/L KCl, 0.2 mg/mL NaN_3_, 20 mmol/L Tris-HCl, pH 7.5; 10:1 buffer-to-meat ratio) at 6,000 rpm for 2 min. After a 15-min incubation at 4 °C, the homogenate was centrifuged at 8,000 rpm for 10 min, and the supernatant was discarded. The resulting pellet was then resuspended in buffer (0.45 mol/L KCl, 20 mmol/L Tris-HCl, 5 mmol/L *β*-mercaptoethanol (*β*-Me), 0.2 mol/L Mg(CH_3_COO)_2_, 1 mmol/L EGTA, pH 6.8; 5:1 buffer-to-pellet ratio). ATP-Na_2_ was added to this suspension to a final concentration of 5 mmol/L. Following a 1 h incubation at 4 °C, centrifugation (10,000 rpm, 10 min) was performed, and the supernatant was collected. This supernatant was diluted with a 6-fold volume of 1 mmol/L KHCO_3_ solution, held at 4 °C for 1 h, and then centrifuged at 12,000 rpm for 10 min. The pellet obtained was homogenized in buffer (0.5 mol/L KCl, 5 mmol/L *β*-Me, pH 7.5; 2.5:1 buffer-to-pellet ratio), incubated at 4 °C for 15 min, and subsequently diluted with a 2.5-fold volume of 1 mmol/L KHCO_3_ solution. Concurrently, MgCl_2_ was added to achieve a final concentration of 10 mmol/L. The homogenate was stored overnight at 4 °C, followed by a final centrifugation step (12,000 rpm, 15 min). The pellet yielded by this final centrifugation was purified myosin. Myosin concentration was determined using Lowry's method [24], with bovine serum albumin as the standard.

#### 2.2.2 HIU treatment and preparation of TGase-catalyzed myosin samples

For HIU treatment, a 0.6 cm diameter titanium probe attached to an ultrasound processor (NingBo Scientz Biotechnology Co. Ltd., Ningbo, China) was used to sonicate 25 mL of samples in 50 mL centrifuge tubes. To prevent heat-induced denaturation, the samples were immersed in an ice-water bath throughout the procedure. To investigate the effects on enzyme physicochemical properties, samples were treated at 150 W (low power) and 400 W (medium power) for 9 min. These specific power levels were selected based on our previous experiments [25], which indicated that excessively high power or prolonged sonication could lead to protein degradation. The HIU intensity was calculated calorimetrically referring to Jambrak et al. [26], yielding values of approximately 25 W·cm^−2^ and 70 W·cm^−2^ for 150 W and 400 W treatments, respectively.

To prepare TGase-catalyzed myosin samples, 10 mL of myosin solution was supplemented with FTGase or MTGase (10 U/g protein), pre-incubated at 37 °C, and subsequently subjected to catalytic reaction at 40 °C for 1 h. Control systems contained enzyme-free myosin. HIU pretreatment was applied to three configurations: (a) isolated enzyme solutions (FTGase or MTGase), (b) myosin-enzyme mixtures, and (c) untreated control. HIU parameters for catalytic reaction in myosin system were optimized at 400 W for 9 min based on the results from the above physicochemical analysis.

### 2.3 Determination of enzyme activity

Enzyme activity was determined according to Hemung & Yongsawatdigul [27] with minor modifications. Briefly, 200 μL of enzyme solution was mixed with 4.4 mL of freshly prepared buffer (62.5 mmol/L Tris, 1.25 mg/mL DMC, 18.75 μmol/L MDC, 3.75 mmol/L dithiothreitol (DTT), 6.25 mmol/L CaCl_2_, pH 7.5) and incubated at 37 °C for 10 min. The reaction was immediately terminated by adding 200 μL of 1 mol/L (NH_4_)_2_SO_4_. Fluorescence intensity was measured at excitation/emission wavelengths of 350/480 nm using enzyme-free buffer as blank. One unit of enzyme activity was defined as the amount catalyzing cross-linking of 1 nmol MDC to DMC per minute, calculated as follows:

$${\text{TGase}}_{\text{activity}}=\frac{({\text{I}}_{\text{S}}\text{-}{\text{I}}_{\text{0}}\text{)}\times {\text{MDC}}_{\text{total}}}{{\text{I}}_{\text{0}}\times \text{t}\times \text{EF}\times {\text{V}}_{\text{enzyme}}}$$

where *I*_S_ and *I*_0_ represent sample and blank fluorescence intensities, respectively; *MDC*_total_ denotes the total MDC content (nmol) in the reaction system; *t* indicates reaction time (min); *EF* corresponds to the fluorescence intensity enhancement factor; and *V*_enzyme_ refers to enzyme volume (mL).

### 2.4 Determination of solubility

Solubility was assessed based on Riebroy et al. [28] with slight modifications. Briefly, 10 mL of enzyme solution was centrifuged at 8,000 rpm for 10 min. The supernatant was collected and subjected to protein determination using Lowry's method [24], with solubility expressed as the percentage of supernatant protein relative to total protein content.

### 2.5 Analysis of UV absorption spectroscopy

UV absorption spectra were recorded according to Wei et al. [29] using a UV2600 spectrophotometer (Shimadzu, Japan). Samples were adjusted to a concentration of 1.0 mg/mL prior to analysis. Scans were performed from 230 to 350 nm at 1 nm intervals.

### 2.6 Molecular docking and molecular dynamics simulations

#### 2.6.1 Homology modeling of FTGase

The structure of FTGase was constructed by homology modeling using Modeller software. The amino acid sequence of carp TGase was retrieved from NCBI (accession number: AAL02240), with TGase 2 (PDB ID: 4PYG) serving as the structural template. The resulting FTGase model underwent 5,000 ps molecular dynamics optimization using AMBER 16 and evaluated via Ramachandran plot analysis using SAVES. The equilibrated structure was selected for subsequent analysis. The derived model of FTGase is presented in Fig. 2A.

#### 2.6.2 Molecular dynamics simulations

To simulate the effect of Ca^2+^ on the structure of FTGase, FTGase was initially solvated in an orthorhombic water box with dimensions of 129 × 83 × 75 Å, followed by the addition of six Ca^2+^. The entire system employed the gaff and ff14SB force fields. Subsequently, the topology and coordinate files were generated with the protein centered in the box. Molecular dynamics simulations were then performed using AMBER16 software, comprising the following steps: (1) two consecutive stages of energy minimization: positional restraints were applied to the protein while minimizing the solvent energy (5000 cycles; initial 1500 cycles using steepest descent algorithm), followed by unrestrained minimization of the entire system (5000 cycles; initial 2000 cycles using steepest descent); (2) system equilibration: temperature equilibration at 300 K was performed for 100 ps using a Langevin thermostat, followed by 100 ps pressure equilibration at 1 bar employing an isotropic Berendsen barostat; (3) production simulation: heavy-atom positional restraints (force constant: 1500 kcal/(mol·Å^2^)) were applied under identical temperature/pressure control to maintain the overall fold while allowing local flexibility. Van der Waals and short-range electrostatic interactions were handled using 10-Å cutoff, while long-range electrostatics were treated with the Particle Mesh Ewald method.

### 2.7 Determination of reactive and total sulfhydryl (−SH) contents

Reactive and total −SH contents were quantified using the Ellman method [30]. For reactive −SH contents, 5.5 mL of sample (1 mg/mL) was reacted with 100 µL of Ellman’s reagent (10 mmol/L DTNB in 0.2 mol/L Tris-HCl buffer, pH 8.0) and incubated at 4 °C for 1 h. Total −SH contents were determined by mixing 0.5 mL of sample (1 mg/mL) with 5 mL of denaturing buffer (8 mol/L urea, 10 mmol/L EDTA, and 2% SDS in 0.2 mol/L Tris-HCl, pH 8.0), followed by the addition of 100 µL of Ellman’s reagent and incubation at 40 °C for 25 min. Absorbance at 412 nm was measured for both assays. −SH contents (*C*_0_, mol/g protein) were calculated as:

$${\text{C}}_{\text{0}}=\frac{\text{A}\times \text{D}}{\text{c}\times \varepsilon }$$

where *A* is the absorbance at 412 nm, *D* denotes the dilution factor, *c* indicates the protein concentration (mg/mL), and *ε* represents the molar extinction coefficient (13,600 L·mol^−1^·cm^−1^).

### 2.8 Analysis of fluorescence spectroscopy

Fluorescence spectra were recorded according to the method described by You et al. [31] with slight modifications. The sample concentration was adjusted to 1 mg/mL. Measurements were performed using a spectrofluorometer (F-4600, Hitachi, Japan) with an excitation wavelength of 295 nm and slit width of 5 nm. Emission spectra from 300 to 450 nm were recorded at a scanning rate of 240 nm/min.

### 2.9 Determination of Ca^2+^-ATPase activity

Ca^2+^-ATPase activity was determined according to Benjakul et al. [9] with some modifications. Briefly, 1 mL of sample (4 mg/mL) was mixed with 0.6 mL of 0.5 mol/L Tris–maleate buffer (pH 7.0), followed by the addition of 1 mL of 0.1 mol/L CaCl_2_. The mixture was brought to 9.5 mL with double-distilled water. The reaction was initiated by adding 0.5 mL of 20 mmol/L ATP solution (pH 7.0), incubated at 25 °C for 10 min, and then terminated by adding 5 mL of 15% (w/w) trichloroacetic acid (TCA). After centrifugation at 5,000 rpm for 5 min, the inorganic phosphate (Pi) content in the supernatant was quantified using the colorimetric method. Ca^2+^-ATPase activity was expressed as μmol Pi/(mg·protein·min). Blank controls replaced the protein solution with equal-volume buffer.

### 2.10 Determination of particle size

The particle size distribution was determined using a laser diffraction particle analyzer (Zetasizer Nano ZS, Malvern Instruments, UK). The measurement parameters were set as follows according to Li et al. [32]: refractive index of the dispersed phase, 1.45; refractive index of the dispersant, 1.33; and viscosity, 0.8873 mPa·s. A 2 mL aliquot of sample solution (1 mg/mL) was loaded into a polystyrene cuvette for analysis at 25 °C. Dynamic light scattering signals were acquired with a 173° non-invasive back-scatter optical configuration. Particle size distribution and average size were automatically calculated by the instrument software (Dispersion Technology Software v4.2) through autocorrelation function analysis.

### 2.11 Determination of non-disulfide covalent bonds

The amount of non-disulfide covalent bonds was assessed by sample solubility in a specific solution (20 mmol/L Tris-HCl, pH 8.0) containing 1% (w/v) SDS, 8 mol/L urea, and 2% (v/v) *β*-Me. Samples (1 g) were homogenized in 20 mL of this solution at 4,000 rpm for 1 min, then heated at 100 °C for 2 min. After magnetic stirring (HJ-3, Guohua Instrument Inc., China) for 4 h, the mixture was centrifuged (4,000 rpm, 30 min). Subsequently, 10 mL of the supernatant was collected and mixed with 2 mL of ice-cold 50% (w/v) TCA and held at 4 °C for 18 h. Following centrifugation (4,000 rpm, 15 min), the pellet was rinsed with 10% (w/v) TCA and dissolved in 0.5 mol/L NaOH. Protein content was quantified using Lowry’s method [24], with solubility (%) expressed as protein content in supernatant to the total protein content.

### 2.12 Determination of WHC

WHC was measured via centrifugation following Chen et al. [33]. Pre-weighed centrifuge tubes (*W*_1_) were loaded with myosin samples, and the combined weight (tube + sample) was recorded as *W*_2_. After centrifugation (4,000 rpm, 10 min), the expelled water was removed. The residual sample-tube weight was designated *W*_3_. All measurements were performed in quintuplicate. WHC was calculated as:

$$\text{WHC/\%=}\frac{{\text{W}}_{\text{3}}\text{-}{\text{W}}_{\text{1}}}{{\text{W}}_{\text{2}}}\times \text{100}$$

### 2.13 Statistical analysis

All experiments were independently repeated three times with at least triplicate measurements per repeat unless otherwise stated. Data were presented as mean ± standard deviation. Figures were generated using Origin 9 (Origin Lab., Northampton, MA, USA). Statistical analysis was performed with SPSS 22 (IBM SPSS Inc., Chicago, IL, USA) through one-way analysis of variance with Duncan's multiple range test. *P* < 0.05 was considered significant difference.

## 3 Results and discussion

### 3.1 Effects of NaCl concentrations on the activity, solubility and structure of FTGase and MTGase

#### 3.1.1 Enzymatic activity

**Fig. 1:**
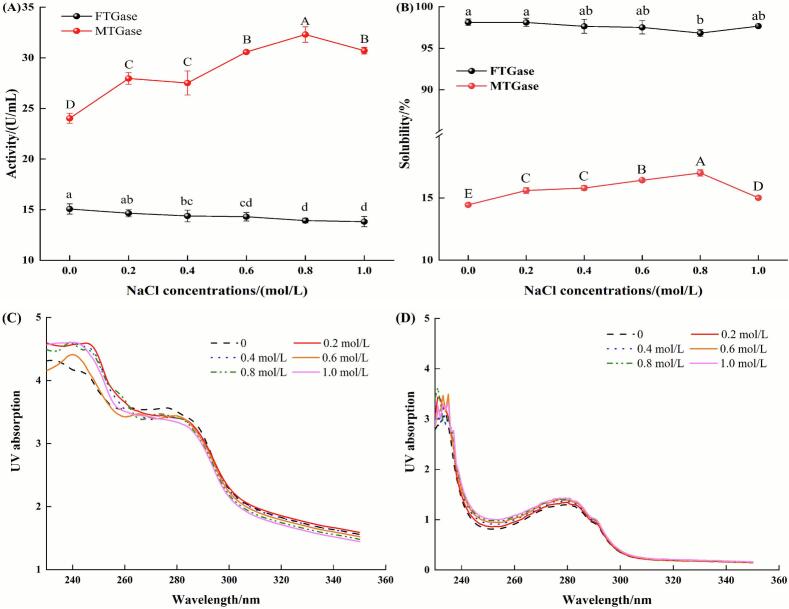
Effects of NaCl concentrations on the enzyme activity (A), solubility (B), UV absorption of FTGase (C), and UV absorption of MTGase (D). Different lowercase letters in (A) and (B) indicate significant differences at the *P* < 0.05 level for FTGase samples. Different capitals in (A) and (B) indicate significant differences at the *P* < 0.05 level for MTGase samples.

The effects of NaCl concentrations on the enzymatic activity of FTGase and MTGase are shown in Fig. 1A. Increasing NaCl concentrations significantly decreased FTGase activity (*P* < 0.05), with activity reduced by 8.3% at 1.0 mol/L NaCl compared to that in the control (without NaCl). Similar inhibition by NaCl was reported for TGase purified from tilapia and Japanese oyster [22,34]. However, the activity of TGase extracted from scallop, botan shrimp, and squid was reported to increase by 11-, 2-, and 6-fold, respectively, in the presence of 0.5 mol/L NaCl [13]. This difference in NaCl response might originate from the environmental habitat of aquatic species. As silver carp is a freshwater fish, the observed sensitivity of FTGase to higher NaCl concentrations might reflect an adaptation to its low-salinity environment.

**Fig. 2:**
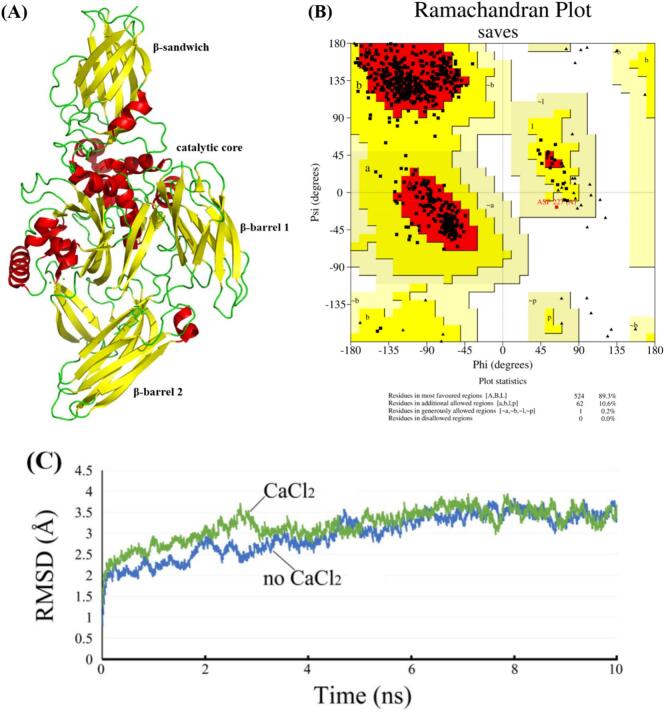
Three-dimensional structure of FTGase from homology modeling (A), Ramachandran plot of FTGase (B), and root mean square deviation (C) of FTGase in the presence or absence of CaCl_2_.

Fig. 1A shows that MTGase activity initially increased and then decreased as NaCl concentrations increased, with peak activity (a 34.5% increase) observed at 0.8 mol/L NaCl. This observation aligned with the findings of Kutemeyer et al. [12], who demonstrated that monovalent cations (Na^+^, K^+^) enhanced MTGase activity and attributed this enhancement to improved enzyme solubility and structural stabilization. However, further investigation is needed to fully elucidate the mechanism. Furthermore, the overall activity level of MTGase was higher than that of FTGase. This difference might derive from the Ca^2+^-independent nature of MTGase, potentially conferring greater inherent stability or activity under the assay conditions used.

#### 3.1.2 Solubility

Fig. 1B illustrates the effects of NaCl concentrations on the solubility of FTGase and MTGase. FTGase maintained consistently high solubility (>95%) across all tested NaCl concentrations, with minimal variation observed. In contrast, MTGase exhibited a distinct solubility profile characterized by an initial increase followed by a decrease, which was consistent with the changes in activity (Fig. 1A). The inherently lower solubility of MTGase compared to FTGase was attributed to its tendency to form metastable colloidal suspensions in aqueous buffers, whereas FTGase was fully water-soluble. The observed increase in MTGase solubility might be due to electrostatic screening effects by Na^+^ and Cl^−^ ions, reducing inter-particle repulsion and promoting colloidal stability [35]. According to the parallel trends between solubility and enzymatic activity, MTGase activity appeared strongly correlated with its solubility, while the activity of FTGase showed minimal correlation with solubility.

#### 3.1.3 Structural changes

To further investigate structural changes in FTGase and MTGase at different NaCl concentrations, UV absorption spectra were analyzed. Both FTGase and MTGase exhibited characteristic absorption peaks near 280 nm (Fig. 1C–D), indicating the presence of aromatic residues (tryptophan, tyrosine, phenylalanine) within their structures. FTGase showed slight alterations in its UV absorption spectra with increasing NaCl concentrations (Fig. 1C), suggesting negligible structural changes. In contrast, MTGase displayed NaCl concentration-dependent enhancement in UV absorption intensity (Fig. 1D), indicating improved solvent exposure of buried aromatic chromophores. This reflected partial unfolding of MTGase. These observed spectral differences between FTGase and MTGase might originate from their structural stability. Given that FTGase is Ca^2+^-dependent, further molecular dynamics simulations were performed to examine Ca^2+^-induced changes in FTGase.

### 3.2 Effects of Ca^2+^ on the structure of FTGase

#### 3.2.1 Homology modeling and structure evaluation

Fig. 2A displays the homology-modeled three-dimensional structure of FTGase, with its Ramachandran plot validation shown in Fig. 2B. The Ramachandran analysis revealed 89.3% of residues in the most favored region and 10.6% in the allowed region (Fig. 2B). This distribution exceeded established stereochemical quality thresholds [36], confirming the structural reliability of FTGase for the subsequent studies. Structurally, FTGase contained four domains: a β-sandwich motif, a catalytic core domain, and two β-barrel domains (β-barrel 1 and β-barrel 2; Fig. 2A). This aligned with the reported TGase structure from *Oncorhynchus mykiss* [37]. Additionally, the catalytic core domain featured both α-helices (red region) and β-sheets (yellow region), while the β-sandwich and the two β-barrel domains exhibited predominantly β-sheet structures.

#### 3.2.2 Molecular dynamics simulations

The root mean square deviation (RMSD) of protein backbone atoms can be employed to evaluate the stability of the complex during molecular dynamics simulations [38]. Fig. 2C shows the RMSD of FTGase in the presence and absence of CaCl_2_. Both systems exhibited an increase in RMSD during the first 3.5 ns, with the Ca^2+^-bound complex displaying slightly higher values than the Ca^2+^-free system. Thereafter, the RMSD stabilized with fluctuations constrained within 3.0 ± 0.2 Å for both systems throughout the remaining simulations, indicative of stable protein structure and maintained integrity.

**Fig. 3:**
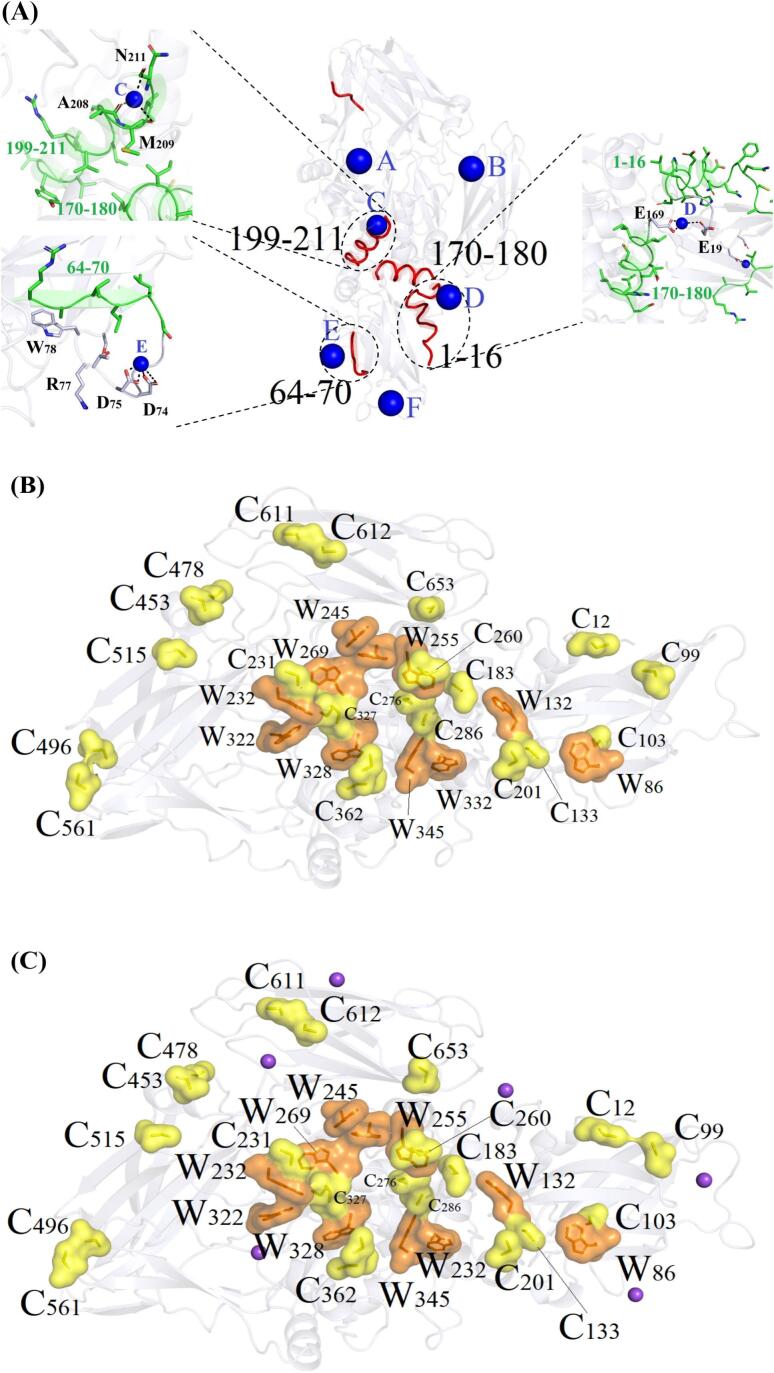
Detailed three-dimensional binding mode of FTGase-Ca^2+^ complex (A), and schematic representation of FTGase structure illustrating the degree of exposure of cysteine and tryptophan residues in the absence (B) and presence (C) of CaCl_2_. Cysteine and tryptophan residues in (B) and (C) are indicated in yellow and orange, respectively. Blue spheres in (A) represent Ca^2+^ ions. Purple spheres in (C) indicate Ca^2+^ ions. (For interpretation of the references to colour in this figure legend, the reader is referred to the web version of this article.)

Spatial analysis was subsequently performed to predict FTGase-Ca^2+^ interactions. Fig. 3A illustrates the three-dimensional binding mode of FTGase with Ca^2+^. Specifically, the Ca^2+^ at site C coordinated with Ala208, Asn211, and Met209, while the Ca^2+^ at site D chelated Glu169 and Glu19. Additionally, site E Ca^2+^ interacted with Asp74 and Asp75. It was speculated that chelation between Ca^2+^ and these negatively charged residues induced FTGase structural changes. Furthermore, Cys272 was established as the catalytic residue in the active triad of rainbow trout FTGase [39]. Our finding demonstrated that the Ca^2+^ at site C engaged with residues adjacent to this catalytic center, suggesting a regulatory role in activating FTGase.

It has been confirmed that the catalytic center of FTGase comprises the Cys-His-Asp triad [40]. Within this center, the catalytically essential cysteine resides in a hydrophobic channel formed by adjacent tryptophan residues. Changes in the solvent accessibility of this cysteine and the surrounding tryptophan residues serve as key indicators for evaluating structural changes in FTGase induced by Ca^2+^ binding. Fig. 3C reveals an increase in the solvent accessibility of these residues upon Ca^2+^ addition compared to the Ca^2+^-free state (Fig. 3B). This suggested that Ca^2+^ induced structural changes within FTGase, potentially leading to increased accessibility of the critical catalytic center.

Considering its Ca^2+^-independent nature and superior activity compared to FTGase, MTGase was selected as the representative enzyme for subsequent studies on the synergistic effects of HIU and NaCl.

### 3.3 Synergistic effects of HIU and NaCl on the activity, solubility and structure of MTGase

#### 3.3.1 Enzymatic activity

**Fig. 4:**
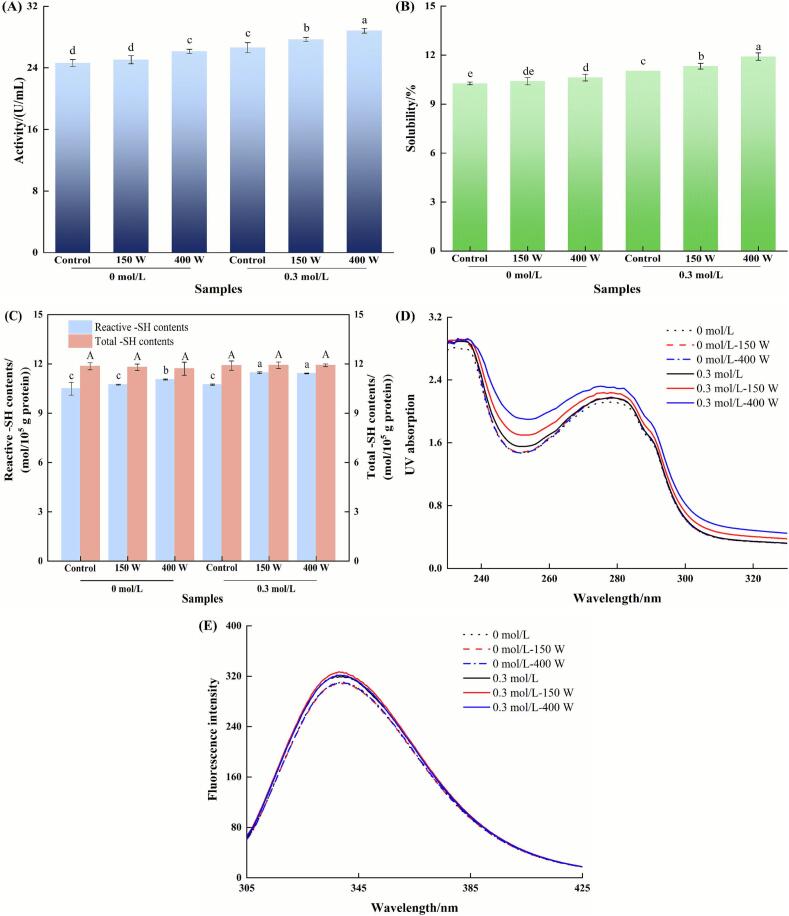
Effects of HIU on the enzyme activity (A), solubility (B), reactive and total −SH contents (C), UV absorption (D), and fluorescence intensity (E) of MTGase. Different lowercase letters in (A) and (B) indicate significant differences at the *P* < 0.05 level. Different lowercase letters in (C) indicate significant differences between reactive −SH contents (*P* < 0.05). Different capitals in (C) indicate significant differences between total −SH contents (*P* < 0.05).

Fig. 4A illustrates the synergistic effects of HIU and NaCl on MTGase activity. In the absence of NaCl (0 mol/L), 150 W HIU exhibited no significant effect on MTGase activity (*P* > 0.05), whereas 400 W HIU increased activity by 6.1% compared to the control (*P* < 0.05). When NaCl concentrations were elevated to 0.3 mol/L, both 150 W and 400 W HIU significantly enhanced MTGase activity by 4.0% and 8.3%, respectively, relative to the 0.3 mol/L control (*P* < 0.05). The cavitation effects generated by HIU, accompanied by mechanical forces (e.g., shear forces and microjets), could alter protein structure and functional properties [41]. Notably, combined treatments of 150 W or 400 W HIU with 0.3 mol/L NaCl increased MTGase activity by 12.4% and 17.0%, respectively, compared to the control groups (0 mol/L NaCl), exceeding the individual effects of either HIU or NaCl alone. Collectively, elevated ionic strength potentiated the HIU-induced enhancement of MTGase activity, confirming synergistic effects.

#### 3.3.2 Solubility

Effects of HIU on the solubility of MTGase at different NaCl concentrations are shown in Fig. 4B. HIU significantly improved MTGase solubility under both NaCl concentrations (0 and 0.3 mol/L) versus their respective controls (*P* < 0.05), except for 150 W HIU at 0 mol/L NaCl. The mechanism involved HIU-induced mechanical effects, such as shear forces and microjets, that disrupted protein aggregates [25], thereby increasing the specific surface area and strengthening protein-solvent interactions to improve solubility. This observation was consistent with reported HIU-mediated solubility enhancements in myosin and soy protein [25,42]. At 0 mol/L NaCl, 400 W HIU increased MTGase solubility by 3.5% over the control. Additionally, 150 W and 400 W HIU combined with 0.3 mol/L NaCl elevated MTGase solubility by 10.2% and 16.0%, respectively, relative to the control without NaCl addition, suggesting an effective synergistic improvement in MTGase solubility by HIU and NaCl.

#### 3.3.3 Reactive and total −SH contents

Fig. 4C depicts the changes in reactive and total −SH contents of MTGase under HIU and NaCl. At 0 mol/L NaCl, 400 W HIU significantly increased reactive −SH content versus the control group (*P* < 0.05). When NaCl concentrations were raised to 0.3 mol/L, 150 W HIU effectively elevated reactive −SH contents (*P* < 0.05). These results indicated that HIU induced structural changes in MTGase more effectively in the presence of NaCl, exposing buried −SH groups and thereby enhancing reactive −SH contents.

It was also evident from Fig. 4C that the total −SH contents remained statistically unchanged across all samples (*P* > 0.05), suggesting no disulfide bond modification occurred. Although HIU could promote disulfide bond formation via radical-mediated oxidation in proteins like myosin and bovine serum albumin [25,43], Lei et al. [44] observed minimal impact on total −SH contents in phosvitin. This discrepancy likely originated from differences in protein structure and HIU parameters.

#### 3.3.4 Structural changes

Further structural changes induced by HIU in the presence and absence of NaCl were characterized by UV absorption and intrinsic fluorescence spectroscopy. As shown in Fig. 4D, all treated MTGase samples exhibited a distinct absorption peak at 280 nm, arising from aromatic amino acids (tryptophan, tyrosine, and phenylalanine). HIU at 150 W or 400 W increased peak absorbance compared to the untreated control, regardless of NaCl presence, indicating protein unfolding. Furthermore, the combination of 400 W HIU and 0.3 mol/L NaCl produced the largest increase in UV absorbance compared with the no-NaCl control, suggesting that NaCl facilitated HIU-driven unfolding and exposure of additional chromophores.

Intrinsic fluorescence spectroscopy (excitation at 295 nm) provided complementary insights into protein structural alterations [45]. Fig. 4E reveals that all samples displayed an emission maximum near 340 nm. While HIU caused minimal shifts in emission wavelength across NaCl levels—implying limited changes in the microenvironment of fluorophores—it obviously modulated fluorescence intensity. In the absence of NaCl, HIU had negligible effects on fluorescence intensity, whereas in 0.3 mol/L NaCl, HIU markedly enhanced fluorescence intensity compared to the control. These results further confirmed that NaCl potentiated HIU-induced unfolding, increasing solvent exposure of tryptophan residues.

### 3.4 Effects of HIU on the aggregation of enzyme-catalyzed myosin samples

#### 3.4.1 Ca^2+^-ATPase activity

**Fig. 5:**
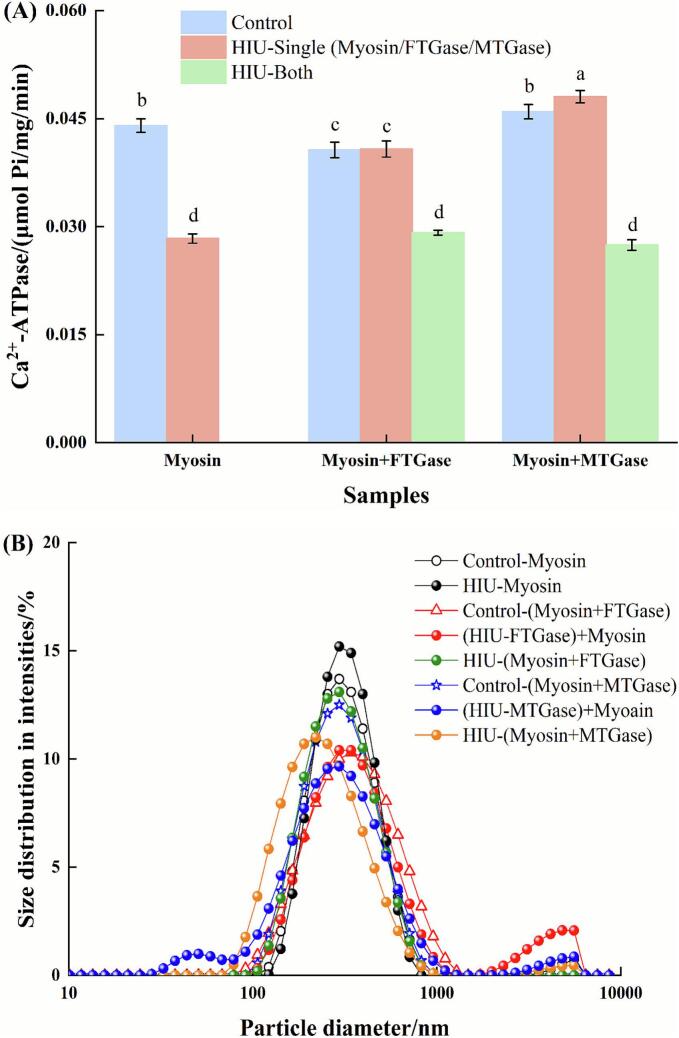
Effects of HIU on the Ca^2+^-ATPase activity (A), and particle distribution (B) of transglutaminase-catalyzed myosin samples. Different lowercase letters indicate significant differences at the *P* < 0.05 level.

Ca^2+^-ATPase activity, a well-established indicator of myosin structural integrity [46], was employed to assess the impact of HIU on enzyme-catalyzed myosin systems (Fig. 5A). In non-HIU processed control groups, the addition of FTGase significantly reduced Ca^2+^-ATPase activity (*P* < 0.05), whereas MTGase exhibited no significant effect (*P* > 0.05). This divergence likely originated from the distinct Ca^2+^ dependencies of FTGase and MTGase, while FTGase required Ca^2+^ for activation, potentially competing with myosin for Ca^2+^, and MTGase was Ca^2+^-independent.

HIU pretreatment of enzymes yielded contrasting outcomes, as FTGase retained its suppressive effect on Ca^2+^-ATPase activity (*P* < 0.05 vs. untreated myosin), while MTGase after HIU treatment significantly enhanced Ca^2+^-ATPase activity (*P* < 0.05), achieving the highest values among all groups. It was speculated that HIU-induced structural changes in MTGase might promote the exposure of latent substrate-binding sites, thereby optimizing its interaction with myosin. This targeted modification could stabilize the myosin head structure, preserving functional integrity.

Conversely, direct HIU treatment to myosin alone severely impaired Ca^2+^-ATPase activity (*P* < 0.05), confirming HIU-induced disruption of the catalytic S1 head domain. Critically, when HIU was applied to preformed enzyme-myosin complexes (FTGase-myosin or MTGase-myosin), Ca^2+^-ATPase activity decreased significantly compared to their non-HIU counterparts (*P* < 0.05). This indicated that under in situ reaction conditions, HIU energy primarily caused structural damage to myosin rather than potentiating enzymatic activity.

#### 3.4.2 Particle size

Fig. 5B displays the particle size distribution of myosin following enzymatic cross-linking under HIU. For pure myosin (control), the particle size distribution exhibited a primary peak within the 100–1,000 nm range and a minor peak near 10,000 nm. This bimodal distribution was consistent with our previous report [47], indicating the inherent propensity of myosin to self-assemble into aggregates of varying sizes. The addition of FTGase prior to HIU shifted the dominant distribution towards larger particle sizes. This observation, combined with relatively low catalytic activity of FTGase, suggested that the heterogeneous aggregates were formed. In contrast, MTGase addition resulted in a slight shift of the distribution towards smaller sizes. The potent catalytic activity of MTGase likely promoted the formation of more uniform, compact aggregates or cross-linked networks, explaining the reduced apparent size distribution.

Pretreatment of FTGase with HIU prior to addition to myosin obviously altered the distribution profile, generating a new prominent peak within the 1,000–10,000 nm range. This pronounced shift toward larger aggregates strongly suggested that HIU pretreatment enhanced the catalytic activity of FTGase, facilitating more extensive cross-linking. Similarly, HIU pretreatment of MTGase also induced a peak at larger sizes (>1,000 nm), but concurrently produced a distinct peak below 100 nm. This bimodal distribution likely reflected both enhanced cross-linking activity leading to large aggregates and potential fragmentation or modification of a sub-population.

Myosin samples subjected solely to HIU treatment (without enzyme) displayed a single, monomodal distribution concentrated between 100–1,000 nm. This obvious narrowing and shift to smaller sizes compared to the untreated control could be attributed to the disruptive effects of ultrasonic cavitation. The associated shear forces, microstreaming, and shock waves generated during HIU effectively fragmented large protein aggregates and denatured structures, yielding a more homogeneous population of smaller particles [25]. In the enzyme-myosin systems studied, HIU consistently demonstrated this dominant disruptive effect, modifying the enzymatic cross-linking processes and resulting in a more uniform size distribution compared to the enzyme-myosin complex without HIU.

### 3.5 Effects of HIU on the chemical bonds of enzyme-catalyzed myosin samples

**Fig. 6:**
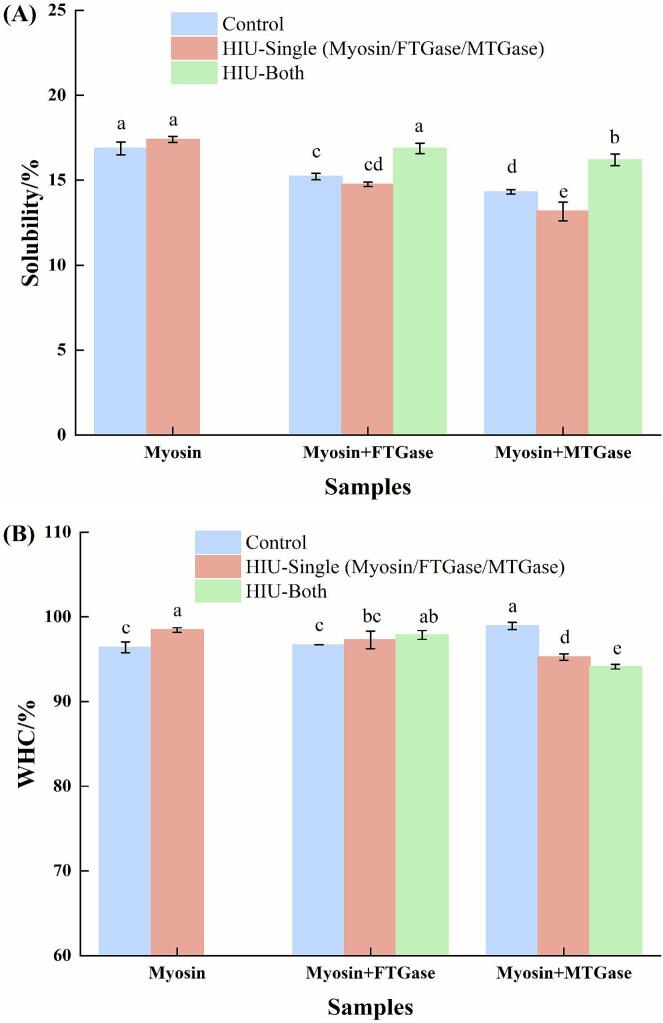
Effects of HIU on the solubility (A) and WHC (B) of transglutaminase-catalyzed myosin samples. Different lowercase letters indicate significant differences at the *P* < 0.05 level.

Solubility can reflect the formation of ε-(γ-Glu)-Lys isopeptide bonds, as the specific buffer used to solubilize samples could disrupt all chemical bonds except for isopeptide cross-linking bonds [11]. As shown in Fig. 6A, the addition of either FTGase or MTGase significantly reduced myosin solubility compared to the untreated control (*P* < 0.05). Crucially, MTGase-treated samples exhibited significantly lower solubility than FTGase-treated samples (*P* < 0.05), indicating more extensive formation of ε-(γ-Glu)-Lys isopeptide bonds. Both enzymes catalyzed the acyl transfer reaction between glutamine and lysine residues, forming ε-(γ-Glu)-Lys bonds. The superior cross-linking efficiency and thus lower solubility observed with MTGase could be attributed to its inherently higher catalytic activity under these conditions, consistent with the activity profiles shown in Fig. 1.

Pretreatment of FTGase or MTGase with HIU prior to addition to myosin significantly decreased solubility compared to the untreated myosin control (*P* < 0.05). While HIU-pretreated FTGase showed a trend towards lower solubility compared to untreated FTGase, this difference was not statistically significant (*P* > 0.05). In contrast, HIU-pretreated MTGase yielded significantly lower solubility than untreated MTGase (*P* < 0.05). This suggested that HIU pretreatment enhanced the catalytic activity of both enzymes, but the activation effect was substantially more pronounced for MTGase.

Solubility of myosin subjected to HIU alone (without enzymes) was not significantly different from untreated myosin (*P* > 0.05). This confirmed that there was little formation of ε-(γ-Glu)-Lys linking bonds in myosin without FTGase or MTGase, even when HIU was applied. For samples where HIU was applied to the preformed enzyme-myosin mixture (i.e., enzyme added before HIU), solubility was significantly increased compared to the corresponding enzyme-treated samples without HIU (*P* < 0.05). This increase in solubility was attributed to the disruptive mechanical forces (cavitation, shear forces) generated by HIU, which could break preformed isopeptide cross-links and/or disrupt the cross-linked protein network. Collectively, these results demonstrated that HIU pretreatment of the enzyme prior to mixing with myosin was a more effective strategy for enhancing ε-(γ-Glu)-Lys cross-linking efficiency (resulting in lower solubility) than applying HIU to the enzyme-myosin mixture.

### 3.6 Effects of HIU on the WHC of enzyme-catalyzed myosin samples

Fig. 6B shows the WHC of myosin subjected to various treatments. Compared to untreated myosin, myosin treated with FTGase did not show a significant alteration in WHC (*P* > 0.05). This was attributed to the inherently low catalytic activity of FTGase, which resulted in insufficient cross-linking to modify the water retention properties of gel networks. In contrast, the addition of MTGase significantly increased WHC compared to both the control and FTGase-treated samples (*P* < 0.05). This enhancement in water retention correlated with the formation of an MTGase-catalyzed protein network, evidenced by a significantly higher level of ε-(γ-Glu)-Lys linking bonds (Fig. 6A).

Pretreatment of FTGase with HIU prior to addition to myosin resulted in WHC not significantly different from either the untreated control or the sample with untreated FTGase (*P* > 0.05). However, pretreatment of MTGase with HIU significantly decreased WHC compared to both the control and the sample with untreated MTGase (*P* < 0.05). Combined with the solubility data (Fig. 6A), which showed the highest level of ε-(γ-Glu)-Lys bonds in this group, this WHC reduction strongly suggested that HIU pretreatment of MTGase induced excessive covalent cross-linking, impairing water holding capacity.

HIU applied directly to myosin (in the absence of enzyme) significantly increased WHC compared to the untreated control (*P* < 0.05). This improvement likely originated from HIU-induced disruption of protein aggregates and structural unfolding, which enhanced protein dispersion and exposed additional polar/hydrophobic groups [11]. These changes facilitated stronger protein–protein and protein-water interactions during subsequent gelation, forming a gel matrix with superior water retention. The results were consistent with observations in soy protein isolate (SPI) systems [48].

For the FTGase-myosin system, HIU significantly improved the WHC compared to untreated control (*P* < 0.05). Combined with the increased solubility (Fig. 6A), the primary mechanism for this WHC increase was likely the HIU-induced dispersion and structural modification of myosin, exposing more water-binding sites rather than significantly enhancing FTGase-mediated cross-linking. Conversely, applying HIU to the preformed MTGase-myosin mixture significantly decreased WHC compared to the corresponding control (*P* < 0.05). This detrimental effect was attributed to the disruptive mechanical forces of HIU breaking preformed isopeptide cross-links (Fig. 6A), thereby compromising its structural integrity and water-holding functionality.

## 4 Conclusion

In the present study, the differential responses of FTGase and MTGase to increasing NaCl concentrations were investigated in terms of activity, solubility and structure. For MTGase, salt-induced structural rearrangements exposed aromatic residues and increased solubility, elevating enzymatic activity. Conversely, FTGase activity declined with increasing NaCl concentrations, most likely because high salt destabilized Ca^2+^-dependent active structure. Based on this, the effect of Ca^2+^ on the structure of FTGase was further investigated. Ca^2+^ could chelate residues (Ala208, Asn211, and Met209) adjacent to the catalytic center, resulting in structural changes in FTGase, thereby enhancing its activity. Considering Ca^2+^-independent properties, MTGase was then selected to explore the synergistic effect of HIU and NaCl. The combination of HIU and 0.3 mol/L NaCl yielded the greatest activity enhancement, driven by HIU/NaCl-induced unfolding. Finally, the catalytic properties of FTGase and MTGase under HIU were investigated using myosin as a substrate. HIU pretreatment of FTGase or MTGase both promoted protein cross-linking, with MTGase showing the larger effect. However, HIU-pretreated MTGase likely induced over cross-linking, leading to decreased WHC. The findings will provide a theoretical basis and a practical strategy for optimizing TGase applications, especially by activating FTGase through HIU or employing MTGase to develop salt-reduced high-quality surimi products with superior texture and water retention.

## CRediT authorship contribution statement

**Xia Gao:** Writing – original draft, Software, Methodology, Investigation, Conceptualization. **Meng Gui:** Validation, Formal analysis. **Gang Yu:** Writing – review & editing, Validation. **Yongqiang Zhao:** Supervision. **Liang Gao:** Data curation. **Ru Liu:** Writing – review & editing, Funding acquisition.

## Declaration of competing interest

The authors declare that they have no known competing financial interests or personal relationships that could have appeared to influence the work reported in this paper.
